# Staging Laparoscopy in High-Risk Gastric Cancer: A Decade of Real-World Evidence and Therapeutic Impact from a Tertiary Referral Center

**DOI:** 10.3390/cancers18010027

**Published:** 2025-12-21

**Authors:** Andrea Cossu, Riccardo Calef, Francesco Puccetti, Silvia Foti, Stefano Cascinu, Riccardo Rosati, Ugo Elmore, OSR CCeR Collaborative Group

**Affiliations:** 1 Department of Gastrointestinal Surgery, IRCCS San Raffaele Scientific Institute, 20132 Milan, Italy; cossu.andrea@hsr.it (A.C.); calef.riccardo@hsr.it (R.C.); rosati.riccardo@hsr.it (R.R.); elmore.ugo@hsr.it (U.E.); 2 School of Medicine, Vita-Salute San Raffaele University, 20132 Milan, Italy; cascinu.stefano@hsr.it; 3 Department of Oncology, IRCCS San Raffaele Scientific Institute, 20132 Milan, Italy; foti.silvia@hsr.it

**Keywords:** gastric cancer, staging laparoscopy, peritoneal metastasis, peritoneal cytology, hyperthermic intraperitoneal chemotherapy (HIPEC), pressurized intraperitoneal aerosol chemotherapy (PIPAC)

## Abstract

Background and Aims: Gastric cancer (GC) remains a leading cause of cancer-related mortality, frequently diagnosed at advanced stages. High-risk features—tumor size ≥ 40 mm, cT3/cT4, nodal involvement, diffuse histology, and Borrmann type III/IV—are associated with peritoneal metastasis (PM). Staging laparoscopy with peritoneal washing (PW) is superior to conventional preoperative imaging modalities, including contrast-enhanced CT, MRI, PET/CT and endoscopic ultrasound, in detecting occult peritoneal disease. In this era of personalized medicine and expanding loco-regional strategies such as cytoreductive surgery (CRS)/Hyperthermic IntraPEritoneal Chemotherapy (HIPEC) and Pressurized IntraPeritoneal Aerosol Chemotherapy (PIPAC), accurate staging is crucial. This study assessed the impact of SL and PW in high-risk GC. Methods: We retrospectively analyzed 113 consecutive high-risk GC patients who underwent SL and PW between 2014 and 2024 at our institution. The primary endpoint was detection of PM or positive cytology (CY+). Secondary endpoints were treatment modification, eligibility for loco-regional therapy, and safety. Results: SL/PW identified PM or CY+ in 26 patients (23%), including 16 with CY+ only. None had radiologic signs of peritoneal disease. SL findings altered treatment in all cases: 21 patients (81%) with Peritoneal Cancer Index (PCI) < 6 underwent induction chemotherapy followed by CRS + HIPEC; 5 patients (PCI > 6) were spared non-therapeutic laparotomy and treated with bidirectional systemic chemotherapy and PIPAC. In 10 patients, systemic therapy was shifted from FLOT (fluorouracil, leucovorin, oxaliplatin, and docetaxel) to FOLFOX (fluorouracil, leucovorin, and oxaliplatin) ± nivolumab. No perioperative complications occurred; all patients were discharged within 24 h without delay in systemic treatment. Conclusions: SL with PW is safe and significantly improves staging accuracy in high-risk GC, enabling personalized therapeutic planning. Routine integration of SL should be considered essential in treatment algorithms to guide systemic and loco-regional strategies.

## Simple Summary

Gastric cancer is often diagnosed in advanced stages, and standard imaging frequently fails to reveal subtle peritoneal spread that can radically change prognosis and treatment goals. Staging laparoscopy enables the direct visualization of the abdominal cavity and cytological assessment of peritoneal fluid, offering diagnostic accuracy beyond noninvasive methods. In this study, we reviewed a decade of experience in patients with clinical features indicating high risk for occult peritoneal disease. Nearly one quarter had previously undetected peritoneal involvement, and these findings modified the therapeutic plan in all cases, guiding the selection of systemic therapy, cytoreductive approaches, or intraperitoneal treatments. These results support routine use of staging laparoscopy to achieve precise staging and more individualized management in high-risk gastric cancer.

## 1. Introduction

Gastric cancer (GC) remains one of the leading causes of cancer-related mortality worldwide, with a significant proportion of cases diagnosed at advanced stages. Despite progress in surgical techniques and systemic therapies, the prognosis for advanced GC—particularly when associated with peritoneal metastasis (PM)—remains poor.

Identifying patients with occult peritoneal dissemination is critical, as PM represents a negative prognostic factor and may significantly alter treatment strategy. Conventional imaging modalities such as computed tomography (CT), magnetic resonance imaging (MRI), and endoscopic ultrasound (EUS) often fail to detect low-volume peritoneal disease or microscopic metastases. Also, CT/PET imaging is unable to reveal peritoneal disease unless advanced features such as nodules or ascites are present. Several new tracers (e.g., FAPI) are currently being studied to improve sensitivity in detecting peritoneal metastases, but further studies are needed [1]. Therefore, staging laparoscopy (SL) combined with peritoneal washing (PW) cytology has gained prominence in the preoperative assessment of GC patients, particularly those at high risk for peritoneal spread. Several clinicopathologic factors have been identified as predictors of peritoneal involvement, including tumor size ≥ 40 mm, T3/T4 staging, nodal positivity (N+), diffuse-type histology according to Lauren classification, and Borrmann type III or IV. These features define a subset of “high-risk” patients who may benefit from more accurate staging with SL and PW to avoid non-therapeutic laparotomy and optimize therapeutic planning.

Despite guideline recommendations, SL remains underutilized in clinical practice. A recent European multicenter study showed that only one-third of patients with locally advanced GC underwent SL, and its absence was associated with higher staging discrepancies, lower rates of systemic treatment, and worse postoperative outcomes [2]. Recent advances in multimodal treatment strategies—including systemic chemotherapy, immunotherapy, and loco-regional therapies such as Pressurized IntraPeritoneal Aerosol Chemotherapy (PIPAC) and Hyperthermic IntraPeritoneal Chemotherapy (HIPEC)—have demonstrated promising results, particularly in selected patients with limited peritoneal involvement. In this evolving context, accurate staging becomes even more relevant to tailor appropriate therapies.

The aim of this study is to evaluate the diagnostic yield and clinical impact of SL and PW in patients with high-risk GC treated in a tertiary referral center, focusing on their role in guiding multimodal treatment strategies and avoiding unnecessary laparotomies.

## 2. Materials and Methods

Study Design and Patient Selection

This is a retrospective observational study conducted at the Gastrointestinal Surgery Unit of IRCCS San Raffaele Scientific Institute, Milan, Italy, a high-volume referral center for gastric cancer. We included all consecutive patients with histologically confirmed gastric adenocarcinoma considered at high risk for peritoneal metastasis (PM) who underwent staging laparoscopy (SL) and peritoneal washing (PW) between June 2014 and February 2024.

Patients were classified as “high-risk” based on at least one of the following criteria:

- Tumor size ≥ 40 mm on imaging or endoscopy;
- cT3 or cT4 stage (based on preoperative imaging);
- cN+ status;
- Lauren diffuse histotype (biopsy-proven);
- Borrmann type III or IV.

After induction chemotherapy, repeat staging laparoscopy was routinely performed prior to surgical intervention to reassess peritoneal disease extent and guide final treatment allocation.

Patients with distant metastasis detected on preoperative imaging, prior abdominal carcinomatosis or prior abdominal malignancies were excluded.

Preoperative Assessment

All patients underwent standard preoperative staging, including thoraco-abdominal contrast-enhanced CT, endoscopy with biopsy, and endoscopic ultrasound when indicated. A multidisciplinary tumor board reviewed each case before indication for SL.

Surgical Procedure: Staging Laparoscopy and Peritoneal Washing

SL was performed under general anesthesia via an open Hasson technique or Veress needle insertion. Pneumoperitoneum was established, and a 30° laparoscope was introduced. A systematic inspection of the abdominal cavity was performed, evaluating all abdominal quadrants, the greater and lesser omentum, Douglas pouch, liver surface, diaphragmatic domes, paracolic gutters, and the mesenteric jejunoileal small bowel.

Peritoneal washing cytology was performed before any manipulation of the tumor by instilling 500 mL of saline solution into the peritoneal cavity (Douglas pouch and subdiaphragmatic spaces). The fluid was aspirated and sent for cytological analysis.

In case of visible peritoneal lesions, biopsies were obtained and sent for histopathological confirmation.

Additionally, the extent of peritoneal carcinomatosis was quantified intraoperatively according to the Peritoneal Cancer Index (PCI) of Sugarbaker [3].

Postoperative Management

Molecular characterization of the tumor was routinely performed on diagnostic biopsy specimens to support tailored therapeutic strategies. This profiling included assessment of HER2 status, microsatellite stability (MSS), PD-L1 expression with Combined Positive Score (CPS), and Claudin 18.2 expression.

All cases were discussed within a multidisciplinary tumor board before the indication for staging laparoscopy and were subsequently re-evaluated after staging laparoscopy to guide individualized treatment planning at each step of the therapeutic pathway.

Outcomes and Statistical Analysis

The primary outcome was the detection rate of peritoneal metastases or positive cytology.

Secondary outcomes included:

- Changes in chemotherapy regimen or treatment strategy following SL;
- Rate of unnecessary laparotomies avoided;
- Eligibility for cytoreductive surgery (CRS) and/or loco-regional treatments (HIPEC or PIPAC);
- Postoperative complication rate following SL.

Categorical variables were expressed as frequencies and percentages. Continuous variables were reported as medians with interquartile ranges (IQRs) or means ± standard deviation (SD), as appropriate. Data analysis was performed using Microsoft Excel (Microsoft Corp, Redmond, WA, USA).

Ethical approval

This retrospective, non-interventional study was conducted in accordance with the Declaration of Helsinki and was approved by the local Institutional Ethics Committee. The requirement for informed consent was waived due to the retrospective nature of the study and the use of anonymized data.

## 3. Results

### 3.1. Patient Characteristics

A total of 113 patients with high-risk gastric adenocarcinoma underwent staging laparoscopy (SL) with peritoneal washing (PW). The mean age was 69 years; 73 patients were male (64.6%) and 40 were female (35.4%). Baseline clinicopathologic characteristics are summarized in Table 1.

All patients were of Italian nationality and European descent.

Key high-risk features included:

- Tumor size ≥ 40 mm in 58 patients (51.3%);
- Clinical stage cT3/T4 in 110 patients (97.3%);
- Radiologic evidence of lymph node involvement (cN+) in all patients (100%);
- Diffuse-type histology in 60 patients (53%);
- Borrmann type III or IV in 85 patients (75.2%).

### 3.2. Staging Laparoscopy Findings

Macroscopic peritoneal metastases (PM) were identified in 10 patients (8.8%) during SL. Positive peritoneal cytology (CY+) was observed in 26 patients (23%), including 16 patients without visible PM. Notably, none of the patients had radiological signs of peritoneal disease on preoperative CT. As a result, all 26 patients (23%) were upstaged based on SL and PW findings.

Importantly, staging laparoscopy was not associated with postoperative morbidity, did not delay the initiation of systemic therapy, and allowed an immediate therapeutic re-stratification in all SL-positive patients.

### 3.3. Impact on Treatment Strategy

Findings from staging laparoscopy (SL) and peritoneal washing (PW) identified peritoneal metastases and/or positive cytology (PM/CY+) in 26 patients (23%), resulting in a modification of the treatment strategy in all cases. Specifically:

- Among the 26 PM/CY+ patients, 21 (81%) had a low PCI (<6) or only CY+ and underwent induction systemic chemotherapy followed by cytoreductive surgery (CRS) and hyperthermic intraperitoneal chemotherapy (HIPEC).
- In 5 patients with a high peritoneal cancer index (PCI > 6), non-therapeutic laparotomy was avoided, and treatment was redirected toward bidirectional systemic chemotherapy combined with PIPAC.
- In 10 patients, SL findings prompted an early change in systemic treatment, with a shift from the standard FLOT regimen to FOLFOX ± nivolumab, reflecting a shift toward immunomodulatory regimens better suited for MSI o PDL1 CPS positive tumors with limited peritoneal disease.

Multidisciplinary reassessment following SL allowed for individualized treatment planning in all cases.

A summary of clinical outcomes and therapeutic decisions influenced by SL and PW is provided in Table 2.

These findings highlight the critical role of SL and PW in the early identification of peritoneal disease and underscore their value in guiding clinical decision-making. By enabling accurate staging and facilitating tailored therapeutic strategies, SL and PW significantly influenced treatment pathways, allowing for appropriate selection of candidates for CRS+HIPEC, avoidance of non-beneficial surgery, and timely adjustment of systemic/bidirectional therapy.

## 4. Discussion

### 4.1. Impact of SL/PW on Therapeutic Strategy

Accordingly, the discussion focuses on how staging laparoscopy findings directly influence downstream therapeutic strategies rather than on the efficacy of these treatments themselves.

Our findings confirm that staging laparoscopy (SL) combined with peritoneal washing (PW) is critical for managing high-risk gastric cancer (GC), particularly in cT3/T4 or diffuse-type tumors. This approach improves staging accuracy, detects occult disease, and prevents non-therapeutic surgery.

In our cohort, SL combined with PW identified occult peritoneal disease in 23% of high-risk GC patients, a rate comparable to previously reported data ranging from 20% to 30% in similar populations [4,5,6]. This underscores the sensitivity of SL/PW in detecting subclinical peritoneal dissemination, which conventional imaging often misses.

The recent multicenter POPEC study demonstrated that positive peritoneal cytology (CY+) is an independent negative prognostic factor and predicts early peritoneal progression, even without visible metastases.

Our results align with the POPEC and CYTO-CHIP trials, where SL identified PM/CY+ in 21–27% of high-risk cases, prompting major therapeutic reclassification [7,8].

SL/PW as a “decision point” is now endorsed by major international guidelines (NCCN, ESMO) for locally advanced GC [9,10].

Moreover, in our series, all the SL-positive patients had a significant change in therapeutic strategy, including access to immunotherapy, bidirectional CT + PIPAC or CRS + HIPEC after systemic treatment with chemotherapy, immunotherapy and/or target agents, confirming the pivotal role of SL in guiding individualized treatment pathways.

Additionally, no complications were observed in our series, reinforcing the favorable safety profile of SL reported in systematic reviews [11]. Notably, SL did not cause any delay in the timely initiation of systemic chemotherapy.

### 4.2. Predictive Utility of SL/PW

While positive peritoneal cytology identifies microscopic peritoneal dissemination, macroscopic assessment during staging laparoscopy remains essential to quantify disease burden through the PCI and to guide selection between curative-intent cytoreduction and alternative loco-regional or palliative strategies.

SL/PW demonstrates high sensitivity for occult peritoneal dissemination. CY+ status alone carries the same negative prognosis as visible PM and qualifies as M1 disease in many classifications [12].

Despite negative cytology, peritoneal fluid may still harbor clinically relevant diagnostic and prognostic information. For example, several molecules, such as CEA, CA125 or IL-17, were identified in literature as marker of peritoneal diffusion of GC in peritoneal fluid [13].

Peritoneal tumour DNA appears to be a promising biomarker for detecting peritoneal micrometastasis, even more sensitive than cytology. Also it may aid in more accurately identifying patients who could benefit from loco-regional chemotherapy and monitor the therapeutic efficacy [14].

Incorporating this emerging techniques, like RT-PCR, ctDNA, and exosomal profiling in peritoneal fluid may further enhance staging precision [15].

### 4.3. Considerations in the Era of Precision Oncology

Only a minority of GC patients benefit from molecular therapies (HER2, PD-L1, MSI-H, Claudin 18.2). The majority are “wild-type” and rely on systemic chemotherapy.

For these patients, loco-regional treatments (HIPEC, PIPAC) associated with CRS represent a vital additional option. SL/PW paves the way for early identification of the best candidates even in the absence of molecular targets [16].

Locoregional therapies have emerged as valuable strategies to improve outcomes in gastric cancer with peritoneal metastases, complementing systemic treatment. By delivering chemotherapy directly into the peritoneal cavity, techniques such as Hyperthermic Intraperitoneal Chemotherapy (HIPEC) and Pressurized Intraperitoneal Aerosol Chemotherapy (PIPAC) increase local drug exposure and reduce systemic toxicity. HIPEC is typically used after complete cytoreduction with curative intent, while PIPAC is being explored in palliative, first-line metastatic, and has been proposed recently as neoadjuvant setting [17]. Careful patient selection is key, with performance status, disease burden, and chemosensitivity guiding treatment decisions.

### 4.4. Role of HIPEC in Disease Control

The relevance of HIPEC in this context lies in the fact that their indication strictly depends on accurate peritoneal staging provided by staging laparoscopy. The phase III GASTRIPEC-I trial demonstrated that adding HIPEC to cytoreductive surgery (CRS) significantly improved disease-free survival (7.1 vs. 3.5 months; *p* = 0.047) and metastasis-free survival (10.2 vs. 9.2 months; *p* = 0.029), although overall survival was not significantly different [18].

In addition, the multicenter CYTO-CHIP study by Bonnot et al. provided further evidence supporting the benefit of HIPEC in this setting. Using a propensity score-matched analysis, the study showed that the combination of CRS and HIPEC was associated with improved overall survival compared to CRS alone (median OS: 18.8 vs. 12.1 months; *p* < 0.001), without increasing postoperative morbidity [8].

Retrospective series from Asia have also confirmed these findings, particularly among patients achieving complete cytoreduction (CC-0), where long-term survival has been reported. The mechanistic rationale for HIPEC lies in the synergy between direct intraperitoneal drug delivery, hyperthermia, and thermal cytotoxicity, which collectively enhance locoregional disease control [19].

### 4.5. Role of PIPAC in Disease Control

PIPAC has emerged as a promising locoregional strategy in the management of gastric cancer with peritoneal metastases across various clinical settings. Its clinical relevance is closely linked to the accurate assessment of peritoneal disease burden provided by staging laparoscopy. In the first-line metastatic bidirectional setting, Alyami et al. reported a median overall survival of 19.1 months in 42 patients with unresectable gastric peritoneal metastases treated with cisplatin/doxorubicin PIPAC in combination with systemic therapy. Notably, 14.3% of these patients were successfully downstaged and subsequently underwent CRS + HIPEC, with a major morbidity rate of only 3.1% [16].

Beyond its therapeutic intent, PIPAC has also shown efficacy in the palliation of malignant ascites, providing symptom relief with minimal morbidity and contributing to an improved quality of life [20].

Furthermore, its potential role in the neoadjuvant setting is currently being investigated in the phase III VERONE trial, which is evaluating bidirectional chemotherapy combined with PIPAC in patients with limited peritoneal disease (PCI ≤ 6), with the goal of increasing resectability and improving survival outcomes [17].

Patient selection remains critical for optimal outcomes. Ideal candidates typically present with ECOG performance status 0–1, low-volume ascites, and evidence of chemosensitive disease. Ongoing research is exploring biomarker-driven stratification to further refine patient selection criteria.

Importantly, staging laparoscopy, peritoneal washing, and PIPAC have been reported to be safe and well tolerated, without delaying systemic therapy [16]. Consistently, in our series, no postoperative complications were observed, and all patients were discharged within 24 h, allowing timely continuation of systemic treatment.

### 4.6. Strengths and Limitations

Strengths: large high-risk cohort managed uniformly over 10 years at a high-volume center with extensive gastric cancer expertise. Multidisciplinary approach and integration with loco-regional treatments enhance clinical relevance.

Limitations: retrospective and single-center; lack of survival analysis data; no molecular profiling of PW samples, though such analysis is planned prospectively. Although survival data were not assessed, treatment individualization and avoidance of futile surgery are expected to confer prognostic benefit, as suggested by recent trials.

Although survival outcomes were not analyzed in the present study, prospective survival analyses are currently planned within an institutional registry, aiming to assess overall and progression-free survival according to SL/PW findings and subsequent treatment strategies. This approach will allow a more robust evaluation of the long-term prognostic impact of staging laparoscopy-guided therapeutic pathways.

All patients were of Italian/European descent, which may limit generalizability of our findings to more diverse populations.

### 4.7. Future Perspectives

Future trials like VERONE and prospective biomarker protocols will define the best patient population who could have the greatest benefit from peritoneal-directed therapies and the optimal integration of systemic and loco-regional therapies.

SL/PW remains a key tool in selecting patients for intensified treatment strategies. The addition of liquid biopsy, peritoneal biomarkers, AI-driven imaging, and intraoperative decision tools promises to further refine treatment algorithms.

## 5. Conclusions

The accurate staging of GC is essential to determine prognosis, treatment-intent and optimal multidisciplinary management. Staging laparoscopy (SL) with peritoneal washing (PW) is a safe, accessible, and highly informative step in the management of high-risk gastric cancer. By uncovering occult peritoneal spread—either macroscopic or cytologic—it enables precise staging, prevents non-therapeutic laparotomies, and directs patients toward the most appropriate systemic or loco-regional therapies.

Our findings confirm its pivotal role in therapeutic stratification, particularly for patients lacking molecular targets, ensuring access to intensified and personalized treatment approaches.

Staging laparoscopy should be seen not just as a diagnostic tool, but as the first therapeutic step in a more effective and rational cancer treatment pathway.

## Figures and Tables

**Table 1:** Clinicopathologic Characteristics of the Study Population. *Clinicopathologic characteristics of patients with high-risk gastric cancer undergoing staging laparoscopy and peritoneal washing. The table summarizes the prevalence of established risk factors for occult peritoneal metastasis in the study cohort*.

Age (Mean ± SD)	69 ± 3 Years
Sex (Male/Female)	73/40
Tumor size ≥ 40 mm	58 (51.3%)
Clinical stage cT3/cT4	110 (97.3%)
Clinical lymph node involvement cN+	113 (100%)
Diffuse Lauren type	60 (53%)
Borrmann type III or IV	85 (75.2%)
CY+	26 (23%)
Macroscopic PM	10 (8.8%)
Postoperative complications after SL	0
Discharge within 24 h	113 (100%)

**Table 2:** Clinical Outcomes and Therapeutic Impact of Staging Laparoscopy. *Impact of staging laparoscopy and peritoneal washing on disease upstaging and treatment strategy. The table highlights how SL/PW findings guided therapeutic reclassification, avoided non-therapeutic laparotomy, and enabled selection of patients for systemic and loco-regional treatments*.

Patients with Positive Peritoneal Cytology (CY+)	26 (23%)
Patients with macroscopic peritoneal metastases (PM)	10 (8.8%)
Patients upstaged due to SL/PW findings	26 (23%)
Treatment strategy modified after SL	26 (23%)
Patients spared non-therapeutic laparotomy (PCI > 6) and redirected toward bidirectional systemic CT and PIPAC	5 (4.4%)
Patients (PCI < 6 or CY+) referred for CRS/HIPEC after CT	21 (18.5)
Postoperative complications	0
Discharge within 24 h	113 (100%)

## Data Availability

The data presented in this study are available on request from the corresponding author due to privacy and/or ethical restrictions.
